# Supplementary material for: The role of informal support systems during illness: A qualitative study of solo self-employed workers in Ontario, Canada
Source: PLoS One. 2024 Mar 13;19(3):e0297770. doi: 10.1371/journal.pone.0297770 (PMC10936821; doi:10.1371/journal.pone.0297770)
Supplement: Attachment — Submitted filename: F_Responses _Reviewer and editors_em.docx [file pone.0297770.s001.docx]

**Addressing Editor’s and Reviewers' Concerns**

We would like to thank the editor and reviewers for their thoughtful feedbacks on our paper and feel that much of the feedback has improved our work. Please see our responses to the comments below.

**Editors’ comments**

Thank you for the review of our paper and advising accordingly. We edited throughout our paper; changes throughout the paper are highlighted with yellow color.

1. Please ensure that your manuscript meets PLOS ONE's style requirements, including those for file naming. The PLOS ONE style templates can be found at

<https://journals.plos.org/plosone/s/file?id=wjVg/PLOSOne_formatting_sample_main_body.pdf> and<https://journals.plos.org/plosone/s/file?id=ba62/PLOSOne_formatting_sample_title_authors_affiliations.pdf>

**Response**: Thank you for the reminder. We formatted the manuscript according to journal’s guidelines.

3) In the ethics statement in the Methods, you have specified that verbal consent was obtained. Please provide additional details regarding how this consent was documented and witnessed, and state whether this was approved by the IRB

**Response**: We are glad to explain further this issue, which has been addressed on page# 8.

(added: Oral informed consent was received before interviews started, documented through the transcriptions, stored in a safe location, and approved by the ethics review committee.)

2) We noticed you have some minor occurrence of overlapping text with the following previous publication(s), which needs to be addressed:

- <https://doi.org/10.1186/s12889-023-15471-8>

- <https://doi.org/10.3390/ijerph19095310>

- <http://dx.doi.org/10.1111/ecc.12746>

In your revision ensure you cite all your sources (including your own works), and quote or rephrase any duplicated text outside the methods section. Further consideration is dependent on these concerns being addressed.

**Response**: These previous publications were cited on the pages# 3-5, where some phrases were left without citations

3) We note that you have indicated that data from this study are available upon request. PLOS only allows data to be available upon request if there are legal or ethical restrictions on sharing data publicly. For more information on unacceptable data access restrictions, please see <http://journals.plos.org/plosone/s/data-availability#loc-unacceptable-data-access-restrictions>.

In your revised cover letter, please address the following prompts:

a) If there are ethical or legal restrictions on sharing a de-identified data set, please explain them in detail (e.g., data contain potentially sensitive information, data are owned by a third-party organization, etc.) and who has imposed them (e.g., an ethics committee). Please also provide contact information for a data access committee, ethics committee, or other institutional body to which data requests may be sent.

**Response:** Thank you for the queries. This study received ethics approval from the University of Waterloo Research Ethics Board (ORE # 42202 on January 7, 2021). Our dataset is available upon the request. As it was a PhD project, nobody other than student and supervising committee has access to datasets. According to the REB, this dataset will be stored for seven years. You can contact the REB of the University of Waterloo at 1-519-888-4567 ext. 36005 or oreceo@uwaterloo.ca.

b) If there are no restrictions, please upload the minimal anonymized data set necessary to replicate your study findings as either Supporting Information files or to a stable, public repository and provide us with the relevant URLs, DOIs, or accession numbers. For a list of acceptable repositories, please see <http://journals.plos.org/plosone/s/data-availability#loc-recommended-repositories>.

We will update your Data Availability statement on your behalf to reflect the information you provide.

**Response:** Please see the above responses for these queries.

4. Please amend either the title on the online submission form (via Edit Submission) or the title in the manuscript so that they are identical.

**Response:** Thank you for the suggestions. We believe that our existing title is identical and edited according to the journal’s guidelines.

5. Please include your full ethics statement in the ‘Methods’ section of your manuscript file. In your statement, please include the full name of the IRB or ethics committee who approved or waived your study, as well as whether or not you obtained informed written or verbal consent. If consent was waived for your study, please include this information in your statement as well.

**Reponses:** This is now addressed in the method section. Please see pages # 6 and 8.

Please review your reference list to ensure that it is complete and correct. If you have cited papers that have been retracted, please include the rationale for doing so in the manuscript text, or remove these references and replace them with relevant current references. Any changes to the reference list should be mentioned in the rebuttal letter that accompanies your revised manuscript. If you need to cite a retracted article, indicate the article’s retracted status in the References list and also include a citation and full reference for the retraction notice.

**Response:** We had no issues related to retracted articles in the reference list.

**Reviewer #1:**

The manuscript investigated the informal support systems used by self-employed individuals during illness/injury and reduction or loss of income. The manuscript provides an overview of existing literature, and the methodology is clearly described, justified and appropriate for the research problem. Findings are presented in a structured way, supported by verbatim from the interviews and is in line with the standard for reporting qualitative research. Conclusions are supported by and aligned with the findings. The findings and conclusions contribute to an important, but often overlooked aspect of entrepreneurship/SE.

Response: Thank you very much for the supportive comments, which are inspiring for us.

The following minor corrections may be considered:
- change format of brackets for citations from ( ) to [ ]

**Response**: We are glad to address your suggestion. We have addressed this issue throughout this article consistently.

- The following lines include the author surnames and date in the citation, instead of only the reference list number:
Lines 83, 84, 114, 328, 332, 333, 335, 336, 338, 341, 385

**Response**: Thank you so much for noticing these issues. We have now addressed these inconsistent citations in your suggested lines, and other lines as well.

- Line 91: Hilbrecht (2016) [15] - also include the reference list number after the date. The same applies to
Line 363, Akram and Maitrot (2022) [58]
- Line 95: ...towards SE'd workers - remove 'a'
- Line 104: ...(36). (37). - both sources [36,37]

**Response**: These issues have been addressed. Thank you for drawing attention to these typographical issues.

- Line 146: ...three key themes.../...three major themes... - Choose either key or major, not both.

**Response:** We corrected this to “three major themes”.

- Line 358: Kim, Longest and Aldrich [47]... - Aldrich's surname was left out.
- Line 391: ...'vicious cycle' of loans. - Consider 'vicious cycle' of debt (instead of loans)

**Response**: We are happy to correct and rephrase the wording according to your suggestions. We have addressed this issue accordingly.

**Reviewer #2:**

This is a very interesting study which addresses the importance of health-related issues for self-employed workers. The study clearly demonstrates the importance of governmental social welfare programs as well as informal social networks for providing health care. One of the issues that I think is crucial is the fact that outside of governmental support it is very difficult to provide health care that is necessary to meet the needs of all individuals in society.

I strongly agree with the authors that social networks that individuals build are indeed important for any person's recovery during health-related issues; however, this is not sufficient for meeting other more complex health problems that can only be addressed by government funded health care systems if a society is serious about keep all individuals healthy. A case and point is the recent pandemic where many governments around the world funded the vaccination of millions of people to prevent massive loss of lives. Without such funding the toll of deaths resulting from the pandemic would have been much greater.

**Response:** Thank you so much for your positive comments and observations. We appreciate and agree with you regarding the point that there is no alternative to government support to ensure a healthy nation. This is a point that we make in our conclusion.
